# Predictive value of controlling nutritional status score in postoperative recurrence and metastasis of breast cancer patients with HER2-low expression

**DOI:** 10.3389/fonc.2023.1116631

**Published:** 2023-07-10

**Authors:** Yue Li, Yue Zhang, Zhaoyue Zhou, Lingmin Shang, Yuanxi Huang, Xiangshi Lu, Shaoqiang Cheng

**Affiliations:** ^1^Department of Breast Surgery, Harbin Medical University Cancer Hospital, Harbin, China; ^2^Department of Medical Oncology, Harbin Medical University Cancer Hospital, Harbin, China

**Keywords:** controlling nutritional status, breast cancer, HER-2 low, recurrence, metastasis, prognosis

## Abstract

**Background:**

To investigate the predictive value of controlling nutritional status (CONUT) score in Postoperative Recurrence and Metastasis of Breast Cancer Patients with HER2-Low Expression.

**Methods:**

The clinicopathological data of 697 female breast cancer patients who pathology confirmed invasive ductal carcinoma and surgery in Harbin Medical University Tumor Hospital from January 2014 to January 2017 were retrospectively analyzed. The relationship between CONUT score and various clinicopathological factors as well as prognosis was evaluated.

**Results:**

Based on the cut-off point of ROC curve, compared with the low CONUT score group, the high CONUT score group had worse 5-year RFS. In subgroup analysis, compared with the low CONUT group, the high CONUT group had worse prognosis at different TNM stages. Univariate and multivariate results showed that the low CONUT score group had better overall survival and recurrence-free survival than the high CONUT group.

**Conclusion:**

CONUT score is an independent predictor of postoperative recurrence and metastasis in HER2-low breast cancer patients. It is may be used as an effective tool to predict the recurrence and metastasis of HER2-low breast cancer.

## Introduction

The latest global cancer statistics show that breast cancer has become the most significant contributor to cancer-related morbidity and mortality among women worldwide (1). It is estimated that there are about 410,000 new cases of breast cancer and 110,000 deaths each year in China, making it the most important health problem for women (2). Many studies have now confirmed that breast cancer is a highly heterogeneous malignancy and that tumor development and progression is not a simple procedure, but a complex process involving multiple factors and stages (3–5). The combination of radiotherapy, chemotherapy, and targeted therapy has significantly improved the overall prognosis of breast cancer patients, but a large proportion of patients still experience local recurrence or distant metastases after the cure, or even death. Tailoring treatment to each patient will improve survival and prognosis (6).

As a result, the study of prognostic predictors of breast cancer has received high priority. Currently, the most commonly used serum tumor markers to predict the prognosis of breast cancer patients include carbohydrate antigen 153 (CA153) and carcinoembryonic antigen (CEA), but their specificity and sensitivity are limited (7, 8). In addition, studies have shown that immunity and inflammation play a crucial role in the development and prognosis of cancer (9). Neutrophil-lymphocyte ratio(NLR), Platelet-lymphocyte ratio(PLR), Prognostic nutritional index (PNI), and Controlling Nutritional Status(CONUT) score are also used to assess the response to adjuvant therapy and prognostic outcome of oncology patients (10–12). In particular, it has been shown that the CONUT score correlates with the prognosis of patients with malignancies such as gastric, lung, colorectal, liver, ovarian, cervical, and renal cell cancers (13–15). The prognostic role of the CONUT score in predicting breast cancer is also gaining attention.HER2-based targeted breast cancer therapy is more precise, and HER2-targeted breast cancer therapy is essential to our clinical practice (16). The 2022 CSCO guidelines classify the molecular staging of breast cancer as HER2-positive, HER2 low expression, or HER2-negative depending on HER2 status (17).

HER2 low-expressing breast cancers account for nearly half of all breast cancers and have unique biological and pathological characteristics (18). An in-depth study of this type of disease is our focus, which is of great importance for clinical guidance. Therefore, finding viable prognostic indicators will not only guide the selection and optimization of treatment options for this particular type of patient and predict the prognosis of the disease but will also fill a gap in the biomarker panel for the prediction of this particular type of breast cancer. Existing studies have not performed CONUT scoring between HER2 subgroups and expressed the prognostic value of specific HER2 low-expressing breast cancer patients. Therefore, this paper examines the predictive value of the control CONUT score in the recurrence of metastasis in patients with HER2 low-expressing breast cancer and the clinicopathological characteristics of the patients.

## Material and methods

### Clinical sample and data collection

A retrospective study was performed on 2156 female breast cancer patients who underwent surgery and were pathologically diagnosed as having invasive ductal carcinoma after surgery from January 2014 to January 2017 in Harbin Medical University Cancer Hospital. Data collected included basic demographic characteristics of patients such as age, body mass index (BMI) and menopausal status, family history of tumors, date of diagnosis of breast cancer, and pathological features. All patients received standard treatment.

Inclusion criteria:

(1) Newly diagnosed female patients who underwent surgery and received regular postoperative treatment
(2) Clinical staging from stage I to III
(3) IHC results were defined as HER2 1+ or 2+ and negative FISH according to ASCO/CAP guidelines at diagnosis
(4) Pathological findings were invasive ductal carcinoma
(5) Not received any anti-HER2 medication
(6) Patients with sufficient detailed clinicopathological data

**Figure 1:**
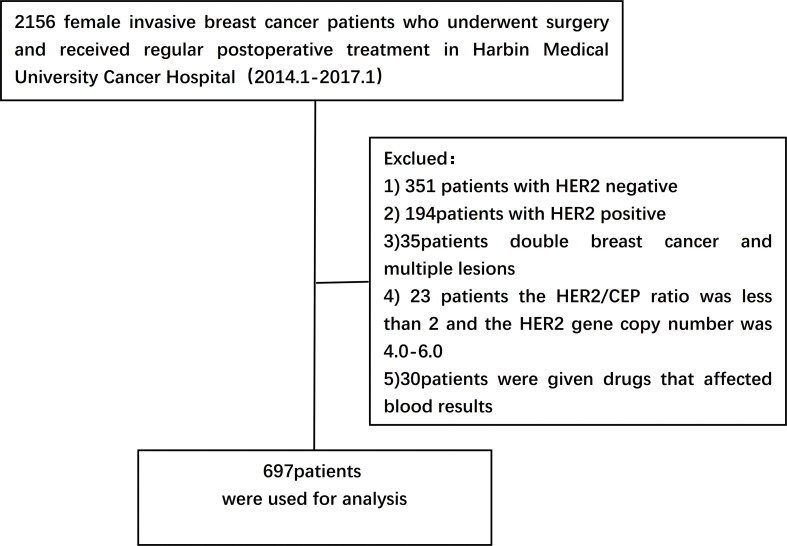
Grouping flow chart of 697 breast cancer patients collected.

Exclusion criteria:

(1) Patients receiving neoadjuvant therapy
(2) Bilateral breast cancer and multiple lesions
(3) HER2/CEP ratio <2 and HER2 gene copy number of 4.0-6.0
(4) Patients who have inflammatory diseases, autoimmune diseases, immune deficiency diseases, infectious diseases or other diseases which affect blood components (such as blood diseases, liver dysfunction, chronic kidney diseases, etc.)
(5) Patients taking drugs that have obvious effects on blood cells(such as Chloramphenicol, Phenobarbital, Chlorpromazine, etc.) (**Figure 1**).

### Definition of data

#### Pathological features and molecular subtypes

Tumor stage according to the AJCC Cancer Staging Manual, Edition 8. ER, PR, HER2 and Ki67 states are assessed by immunohistochemistry (IHC) staining or *in situ* hybridization (ISH), and ER and PR nuclear ≥1% are defined as positive. HER2 immunohistochemical staining was divided into HER2 positive, HER2-low expression and HER2 negative. IHC 3+ or IHC 2+ and ISH positive was defined as HER2-positive;IHC 2+ and ISH negative or IHC 1+ was defined as HER2-low expression;IHC 0 was defined as HER2-negative (19–21). Ki-67 positive nuclear ≥14% was defined as high expression, <14% as low expression (22). Tumor markers CEA and CA153 were considered positive if they exceeded the upper limit of the normal range.

#### Controlling nutritional status score

All patients had to fast peripheral venous blood collected early in the morning within two days of admission, and preoperative serum albumin (g/L), peripheral blood lymphocyte count (×10^9^cells/L) and total serum cholesterol (mmol/L) data were collected retrospectively to calculate the CONUT score. The specific criteria for calculating the CONUT score are as follows: (1) Serum albumin was graded as ≥35.0 g/L, 30.0 ~ 34.9 g/L, 25.0 ~ 29.9 g/L and <25.0 g/L, with scores of 0, 2, 4 and 6, respectively;(2) Total peripheral blood lymphocytes were calculated as ≥ 1.60× 10^9^/L, 1.20~1.59×10^9^/L, 0.80~1.19×10^9^/L and <0.80×10^9^/L, respectively, with scores of 0, 1, 2 and 3;(3) Total serum cholesterol was scored as 0, 1, 2 and 3 in four classes: > 4.68 mmol/L, 3.64~4.68 mmol/L, 2.6~3.63 mmol/L and < 2.6 mmol/L, respectively. The scores of the above three parameters were added together to calculate the total CONUT score of the patients, where 0~1 is a normal, 2~4 is a mild abnormality, 5~8 is a moderate abnormality, and 9 and above is a severe abnormality (**Table 1**).

**Table 1:** Controlling nutritional status index score.

Parameter	Normal	Mild	Moderate	Severe
Total lymphocyte count (*10^9^/L) [score]	≥1,60 [0]	1.2-1.59 [1]	0.8-1.19 [2]	<0.8 [3]
Albumin (g/L) [score]	≥35.0 [0]	30.0–34.9 [2]	25.0–29.9 [4]	<25.0 [6]
Total cholesterol (mmol/L) [score]	> 4. 68 [0]	3. 64~4.68 [1]	2. 6 ~ 3.63 [2]	< 2. 6 [3]
Total score	0-1	2-4	5-8	9-12

### Follow-up and outcome

The survival status and treatment information of all patients were obtained through telephone follow-up and outpatient review in Harbin Medical University Cancer Hospital. The primary endpoint of this study was recurrence free survival (RFS), which was defined as the period from the date of surgery to the date of disease recurrence and metastasis or the end of follow-up. The status of recurrence and metastasis (including local recurrence, regional recurrence, isolated recurrence and distant metastasis) of patients was mainly judged by imaging examination (breast ultrasound, molybdenum target, chest CT, bone scan, etc.) or pathological results of tissue biopsy. The secondary endpoint was overall survival (OS), defined as the time from the date of surgery to any date of death or the date of the last follow-up. The follow-up period was until December 30, 2021.

### Statistics and analysis

Statistical analysis was performed using SPSS 26.0 software. The optimal cut-off point of the CONUT score for predicting recurrence, metastasis and death was determined by the receiver operating characteristics curve (ROC curve) and maximum Youden index. CONUT score and clinicopathological parameters of breast cancer patients were evaluated by Pearson’s χ^2^ test or Fisher exact test method. Kaplan-Meier method was used to draw a survival curve. The Cox regression model was used to conduct univariate and multivariate analysis on the relationship between clinicopathological features and patients’ OS and RFS. *P*<0.05 for a statistically significant difference.

## Results

### Relationship between CONUT score and baseline characteristics of patients

A total of 1216 women with breast cancer after surgery was finally eligible, of which 697 (57.3%) were confirmed to have HER2-low expression and were included in this study. The mean age of the patients was (52.0 ± 9.5) years The median age was 52 years (range: 19-79 years). The age of enrolled patients was divided into the <52 years old group and the ≥52 years old group. Total mastectomy was performed in 93.7%(653/697)patients. 104(19.4%)patients had no history of breastfeeding; 53.8%(375/697)were menopausal at the time of diagnosis; about 39% were overweight, of whom 6% were severely obese. There were 428(61.4%) patients in stage T1, 240(34.4%)patients in stage T2, and 29(4.2%)patients in stage T3; the positive rate of lymph nodes was 45.7%(319/697); The postoperative TNM stage was 269 (38.6%) for stage I patients, 303 (43.5%) for stage II patients and 125 (17.9%) for stage III patients; 546 (78.3%) for histological grade I-II and 152 (21.7%) for grade III; 577 (82.8%) for ER positive patients and 545 (78.2%) for PR positive patients. There were 118 cases of triple-negative breast cancer, accounting for 16.9% of all patients, 282 cases of Luminal A breast cancer (40.5%), and 297 cases of Luminal B breast cancer (42.6%). The baseline characteristics of the patients are shown in **Table 2**.

**Table 2:** Relationship between CONUT score and basic characteristics of patients.

Characteristics	Total (n=697)	CONUT SCORE		χ^2^/Fisher	*p-*value
		Low (n=515)	High (n=182)		
Age (years)				0.055	0.815
<52	328 (47.1%)	241 (34.6%)	87 (12.5%)		
≥52	369 (52.9%)	274 (39.3%)	95 (13.6%)		
Reproductive history				1.649	0.199
Yes	660 (94.7%)	491 (70.4%)	169 (24.3%)		
No	37 (5.3%)	24 (3.4%)	13 (1.9%)		
Breastfeeding history				2.743	0.098
Yes	593 (85.1%)	445 (63.8%)	148 (21.3%)		
No	104 (14.9%)	70 (10.0%)	34 (4.9%)		
Menopause status				0.724	0.395
Yes	375 (53.8%)	282 (40.6%)	93 (13.2%)		
No	322 (46.2%)	233 (33.4%)	89 (12.8%)		
BMI (kg/m^2^)				1.007	0.604
<25	425 (61.0%)	319 (45.8%)	106 (15.2%)		
25-30	230 (33.0%)	167 (23.9%)	63 (9.1%)		
≥30	42 (6.0%)	29 (4.2%)	13 (1.8%)		
CEA (ng/mL)				8.863	0.003
Negative	629 (90.2%)	475 (68.1%)	154 (22.1%)		
Positive	68 (9.6%)	40 (5.7%)	28 (3.9%)		
CA153				5.087	0.024
Negative	631 (90.5%)	473 (67.9%)	158 (22.6%)		
Positive	66 (9.5%)	42 (5.9%)	24 (3.6%)		
NLR				7.579	0.006
<1.88	356 (51.1%)	279 (40.0%)	77 (11.1%)		
≥1.88	341 (48.9%)	236 (33.9%)	105 (15.0%)		
Histological grade				1.360	0.244
1-2	546 (78.3%)	409 (58.6%)	137 (19.7%)		
3	151 (21.7%)	106 (15.2%)	45 (6.5%)		
Surgery type				0.030	0.862
BCS	44 (6.3%)	33 (4.7%)	11 (1.6%)		
Mastectomy	653 (93.7%)	482 (69.2%)	171 (24.5%)		
KI67 status				0.323	0.250
≤14%	270 (38.7%)	193 (27.7%)	77 (11.0%)		
>14%	427 (61.3%)	322 (46.2%)	105 (15.1%)		
ER status				1.675	0.196
Negative	120 (17.2%)	83 (11.9%)	37 (5.3%)		
Positive	577 (82.8%)	432 (62.0%)	145 (20.8%)		
PR status				0.004	0.948
Negative	152 (21.8%)	112 (16.1%)	40 (5.7%)		
Positive	545 (78.2%)	403 (57.8%)	142 (20.4%)		
pT Stage				9.773	0.008
1	428 (61.4%)	326 (46.8%)	102 (14.6%)		
2	240 (34.4%)	163 (23.4%)	77 (11.0%)		
3	29 (4.2%)	16 (2.3%)	13 (1.9%)		
pN stage				38.059	<0.001
0	378 (54.3%)	307 (44.1%)	71 (10.2%)		
1	196 (28.1%)	142 (20.4%)	54 (7.7%)		
2	83 (11.9%)	43 (6.2%)	40 (5.7%)		
3	40 (5.7%)	24 (3.4%)	16 (2.3%)		
pTNM stage				21.737	<0.001
I	269 (38.6%)	213 (30.6%)	56 (8.0%)		
II	303 (43.5%)	230 (3.0%)	73 (10.5%)		
III	125 (17.9%)	72 (10.3%)	53 (7.6%)		
Molecular subtype				4.992	0.082
Luminal A	282 (40.5%)	209 (30.0%)	73 (10.5%)		
Luminal B	297 (42.6%)	228 (32.7%)	69 (9.9%)		
TNBC	118 (16.9%)	78 (11.2%)	40 (5.7%)		
Chemotherapy				3.372	0.066
Yes	574 (82.4%)	416 (59.7%)	158 (22.7%)		
No	123 (17.6%)	99 (14.2%)	24 (3.4%)		
Endocrine therapy				3.480	0.062
Yes	533 (76.5%)	403 (57.8%)	130 (18.7%)		
No	164 (23.5%)	112 (16.1%)	52 (7.4%)		
Radiotherapy				14.737	<0.001
Yes	175 (25.1%)	110 (15.8%)	65 (9.3%)		
No	522 (74.9%)	405 (58.1%)	117 (22.4%)		

**Figure 2:**
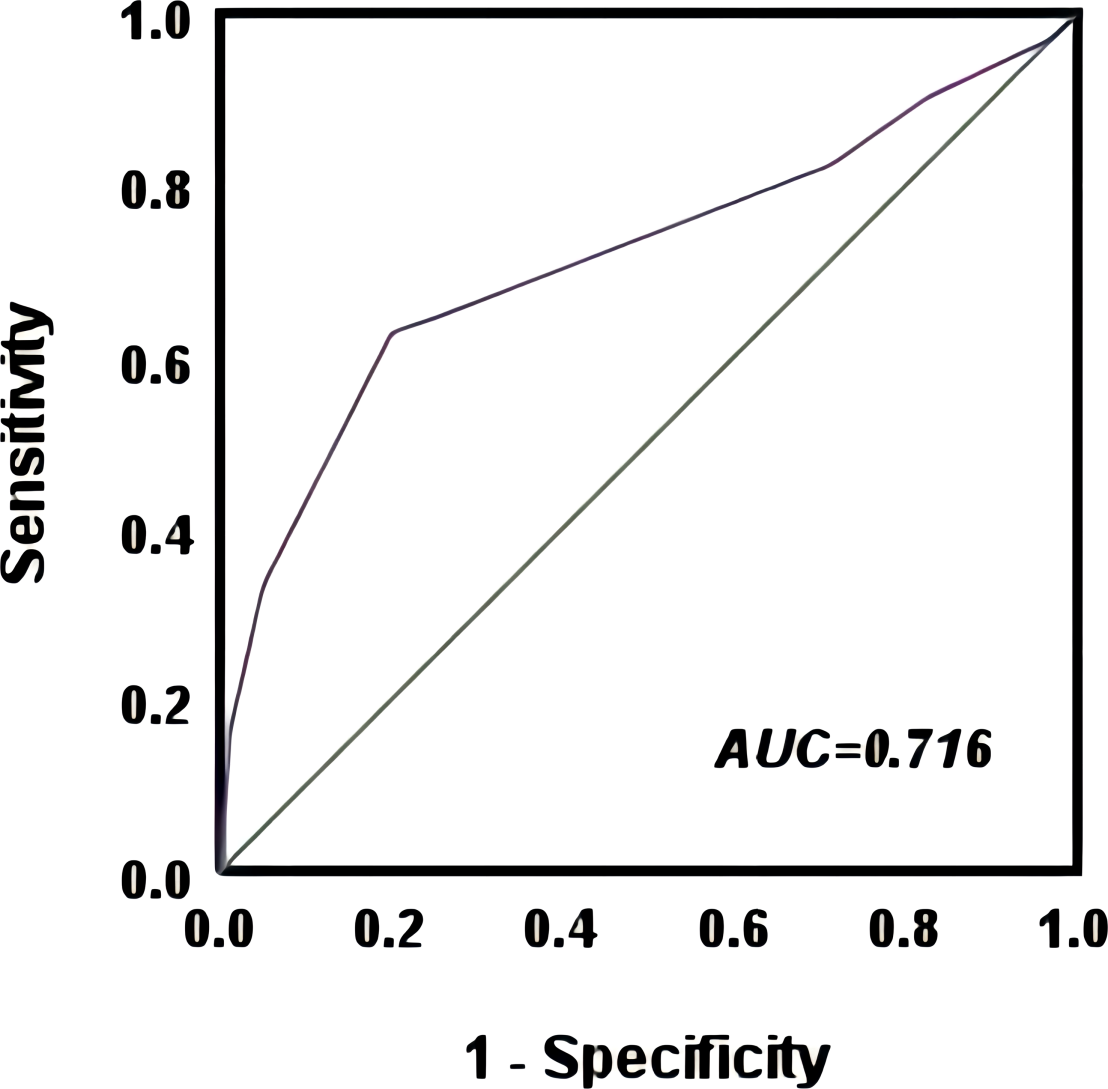
ROC curves for CONUT scores to predict survival in breast cancer patients.

The optimal cut-off point for the CONUT score to predict postoperative recurrent metastasis in breast cancer patients was 2.5 with an AUC value of 0.716 (95% CI=0.660~0.771), as shown by the ROC curve. Based on this cut-off point, all patients were divided into a high CONUT group (≥3 points) and a low CONUT group (<3 points) (**Figure 2**) The results showed that CEA (χ^2^ = 8.863, *P*=0.003), CA153 (χ^2^ = 5.087, *P*=0.024), NLR(χ^2^ = 7.579, *P*=0.006), T-stage (χ^2^ = 9.773, *P*=0.008), lymph node infiltration (χ^2^ = 17.128, *P*=0.001), TNM stage (χ^2^ = 21.737, *P*<0.01), radiotherapy (χ^2^ = 14.737, *P*<0.01), and possibly CONUT high subgroup breast cancer were influential factors (**Table 2**).

### Relationship between CONUT score and recurrence and metastasis in HER2-low breast cancer

**Figure 3:**
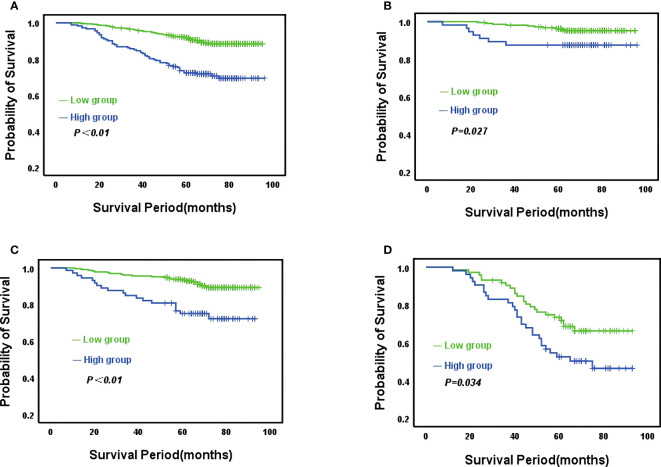
Kaplan-Meier analysis predicts recurrent metastases in HER2-low breast cancer patients based on CONUT score **(A)** All breast cancer patients; **(B)** Stage I breast cancer patients; **(C)**, Stage II breast cancer patients; **(D)** Stage III breast cancer patients.

The median follow-up time for the whole group was 70 months (range: 9-96 months),107 (15.4%) patients developed recurrent metastases during the follow-up period, with RFS rates of 93.2%, 86.8% and 85.9% at 3, 5 and 7 years respectively. Kaplan-Meier curves demonstrated a significantly higher rate of recurrence and metastasis in the high CONUT subgroup compared to the low CONUT subgroup, with 5-year RFS rates of 78.9% and 89%, respectively, with statistically significant differences (χ^2^ = 38.384, *P*<0.01) (**Figure 3A**). Subgroup analysis further analyzed the prognosis of breast cancer patients in the CONUT score subgroup at different stages and showed that the TNM stage was a poor prognostic factor for breast cancer patients in the high CONUT subgroup. For stage I patients, the 5-year RFS rates in the high CONUT group and low CONUT group were 87.5% and 95.3% respectively, with a statistically significant difference (χ^2^ = 4.882, *P*=0.027)(**Figure 3B**); For stage II patients, the 5-year RFS rates in the two groups were 75.4% and 91.3% respectively, with a statistically significant difference (χ^2^ = 14.465, *P*<0.01) (**Figure 3C**); For stage III patients, the 5-year RFS rates for the two groups were 52.8% and 73.9%, respectively, with statistically significant differences (χ^2^ = 4.495, *P*=0.034) (**Figure 3D**).

**Table 3** further analyzes the univariate and multivariate analysis affecting RFS, and the results show that the CONUT score is predictive of recurrent metastasis in breast cancer patients. Multivariate Cox regression analysis showed that lymph node metastasis (HR=3.105, 95%CI=1.998-4.826, *P*<0.001), tumor size (HR=0.543 95%CI=0.349-0.845, *P*=0.007), Ki67 status (HR=2.058, 95%CI=1.277-3.317, *P*=0.003) and CONUT score (HR=2.591, 95%CI=1.736-3.809, *P*<0.001) were independent predictors of recurrent metastasis in patients with early to mid-stage HER2 low expression breast cancer receiving regular treatment. The results showed that the CONUT score had a predictive effect on recurrence and metastasis in breast cancer patients (**Table 3**).

**Table 3:** Univariate and multivariate analyses of recurrence free survival.

Parameters	Univariate analysis	*P* value	Multivariate analysis	*P* value
	Hazard ratio (95%CI)		Hazard ratio (95%CI)	
Age (years)		0.220		
<52	1 (reference)			
≥52	1.272 (0.866-1.870)			
BMI (kg/m^2^)		0.110		
<25	1 (reference)			
≥25	1.364 (0.932-1.995)			
CEA		0.106		
Negative	1 (reference)			
Positive	1.613 (0.903-2.881)			
CA153		0.739		
Negative	1 (reference)			
Positive	0.895 (0.467-1.717)			
ER status		0.811		0.308
Negative	1 (reference)		1 (reference)	
Positive	0.941 (0.573-1.545)		2.090 (0.507-8.627)	
PR status		0.164		0.185
Negative	1 (reference)		1 (reference)	
Positive	0.736 (0.478-1.133)		1.312 (0.878-1.961)	
KI-67		<0.001		0.003
<14%	1 (reference)		1 (reference)	
≥14%	2.237 (1.429-3.501)		2.058 (1.277-3.317)	
Histological grade		0.008		0.092
G1/2	1 (reference)		1 (reference)	
G3	1.749 (1.160-2.636)		1.504 (0.936-2.416)	
TNBC		0.748		0.972
No	1 (reference)		1 (reference)	
Yes	1.085 (0.661-1.781)		0.974 (0.221-4.295)	
Tumor size		<0.001		0.007
≤ 2	1 (reference)		1 (reference)	
>2	2.137 (1.459-3.131)		0.543 (0.349-0.845)	
Lymphatic metastasis		<0.001		<0.001
No	1 (reference)			
Yes	3.669 (2.384-5.647)		3.105 (1.998-4.826)	
CONUT score		<0.001		<0.001
<3	1 (reference)		1 (reference)	
≥3	3.112 (2.130-4.547)		2.591 (1.736-3.809)	
NLR		<0.001		0.002
<1.88	1 (reference)		1 (reference)	
≥1.88	2.199 (1.468-3.293)		1.912 (1.266-2.888)	

### Association between CONUT score and overall survival in HER2-low breast cancer

**Figure 4:**
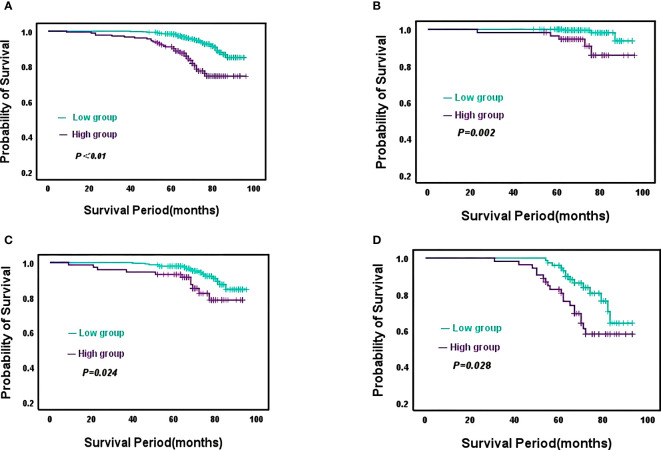
Kaplan-Meier analysis predicts overall survival in HER2-Low breast cancer patients based on CONUT score **(A)**, All breast cancer patients; **(B)**, Stage I breast cancer patients; **(C)**, Stage II breast cancer patients; **(D)**, Stage III breast cancer patients.

A total of 68 (9.8%) deaths occurred during the follow-up period, with overall patient survival rates of 99.2%, 96.5%, and 90.5% at 3, 5, and 7 years respectively. Kaplan-Meier curves demonstrated significantly higher mortality in the high CONUT group compared to the low CONUT group, with 5-year OS rates of 87.9% and 92.5%, respectively, with statistically significant differences (χ^2^ = 26.3215, *P*<0.01) (**Figure 4A**); Subgroup analysis further analyzed the prognosis of breast cancer patients in the CONUT score subgroup at different stages and showed that the TNM stage was a poor prognostic factor for breast cancer patients in the high CONUT subgroup.

For stage I patients, the 5-year OS rates for the high CONUT group and low CONUT group were 96.4% and 98.5%, respectively, with a statistically significant difference (χ^2^ = 9.200, *P*=0.002)(**Figure 4B**); For stage II patients, the 5-year OS rates for the two groups were 93.1% and 96.0%, respectively, with statistically significant differences (χ^2^ = 5.088, *P*=0.024) (**Figure 4C**); For stage III patients, the 5-year OS rates for the two groups were 80.6% and 90.1%, respectively, with a statistically significant difference (χ^2^ = 4.845, *P*=0.028) (**Figure 4D**).

**Table 4** incorporates relevant clinicopathological variables for further univariate and multivariate analyses affecting OS, and the results indicate that the CONUT score is also predictive of overall survival in breast cancer patients. Multivariate Cox regression analysis showed that radiotherapy (HR=2.111, 95%CI=1.243~3.587, *P*=0.006), lymph node infiltration (HR=3.064, 95%CI=1.578-5.950, *P*=0.001), Ki67 status (HR=1.896, 95%CI=1.041-3.453, *P*=0.036) and CONUT score (HR=2.520, 95%CI=1.549-4.099, *P*<0.001) were independent factors affecting the survival prognosis of patients with HER2 low expression breast cancer. The above findings further illustrate the feasibility of the CONUT score in predicting survival and recurrence in breast cancer patients.

**Table 4:** Univariate and multivariate analyses of overall survival.

Parameters	Univariate analysis	*P* value	Multivariate analysis	*P* value
	Hazard ratio (95%CI)		Hazard ratio (95%CI)	
Age (years)		0.915		
<52	1 (reference)			
≥52	1.026 (0.637-1.054)			
BMI (kg/m^2^)		0.690		
<25	1 (reference)			
≥25	1.103 (0.680-1.789)			
CEA		0.313		
Negative	1 (reference)			
positive	1.462 (0.699-3.057)			
CA153		0.733		
Negative	1 (reference)			
positive	0.864 (0.374-1.998)			
ER status		0.489		0.479
Negative	1 (reference)		1 (reference)	
Positive	0.813 (0.451-1.463)		2.048 (0.281-14.949)	
PR status		0.437		0.509
Negative	1 (reference)		1 (reference)	
Positive	0.807 (0.471-1.385)		0.820 (0.456-1.475)	
KI-67		0.008		0.036
<14%	1 (reference)		1 (reference)	
≥14%	2.096 (1.210-3.632)		1.896 (1.041-3.453)	
Histological grade		0.032		0.442
G1/2	1 (reference)		1 (reference)	
G3	1.760 (1.051-2.945)		1.265 (0.695-2.303)	
TNBC		0.467		0.594
No	1 (reference)		1 (reference)	
Yes	1.244 (0.691-2.239)		1.765 (0.219-14.249)	
Tumor size		0.003		0.299
≤ 2	1 (reference)		1 (reference)	
>2	2.100 (1.298-3.397)		1.311 (0.787-2.183)	
Lymphatic metastasis		<0.001		0.001
No	1 (reference)		1 (reference)	
Yes	4.668 (2.593-8.403)		3.064 (1.578-5.950)	
Chemotherapy		0.013		0.905
No	1 (reference)		1 (reference)	
Yes	3.604 (1.312-9.898)		1.068 (0.361-3.165)	
Radiotherapy		<0.001		0.011
No	1 (reference)		1 (reference)	
Yes	6.486 (2.535-16.598)		2.007 (1.173-3.433)	
Endocrine therapy		0.431		
No	1 (reference)			
Yes	0.808 (0.476-1.373)			
CONUT score		<0.001		<0.001
<3	1 (reference)		1 (reference)	
≥3	3.242 (2.014-5.219)		2.520 (1.549-4.099)	
NLR		0.026		0.163
<1.88	1 (reference)		1 (reference)	
≥1.88	1.753 (1.071-2.871)		1.429 (0.865-2.360)	

### Relationship between ER status and CONUT scores

We analyzed the relationship between strong ER positivity and CONUT scores. Using 10% as the cut-off point for strong ER positivity, 513 of 697 patients were strongly ER-positive, of which 129 were in the high CONUT group and 384 were in the low CONUT group. Strongly positive ER(χ^2^ = 1.884, *P*=0.170) by chi-squared test, not statistically significantly different from the CONUT group.

**Figure 5:**
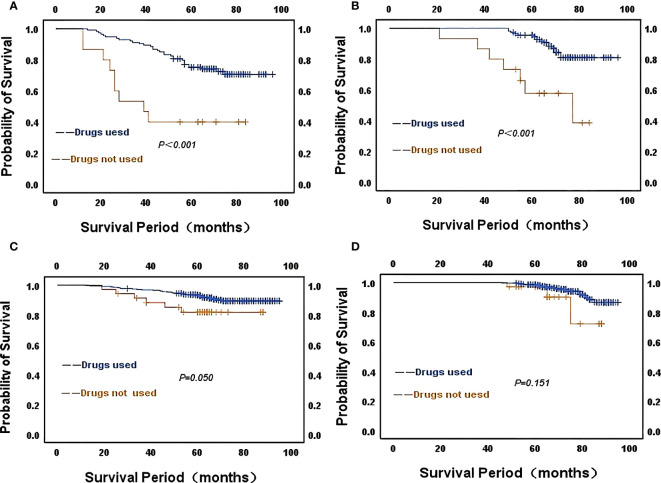
Kaplan-Meier survival curves of strongly ER-positive patients in different CONUT score groups about the use of endocrine drugs **(A)** CONUT high score for RFS; **(B)** CONUT high score for OS; **(C)** CONUT low score for RFS; **(D)** CONUT low score for OS.

However, we analyzed the relationship between ER strongly positive patients in different CONUT score groups and the use of endocrine drugs by Kaplan-Meier survival curves. 114 patients in the high CONUT subgroup used endocrine drugs, and RFS (χ^2^ = 13.606, *P*<0.001) and OS (χ^2^ = 16.151, *P*<0.001) were statistically different. Thirty-five patients in the low CONUT group were not on endocrine medication and although there was no statistically significant difference in RFS(χ^2^ = 3.841, *P*=0.050) and OS(χ^2^ = 2.060, *P*=0.151), the curves changed significantly. It was concluded from the Kaplan-Meier survival curves that patients using endocrine drugs had longer RFS and OS than those not using endocrine drugs, both in the high CONUT subgroup and in the low CONUT subgroup (**Figure 5**).

Kaplan-Meier survival curves to statistically analyze the duration of endocrine drug use in patients in different CONUT score groups. Of the 114 patients using endocrine drugs in the CONUT high subgroup, 63 patients had been on endocrine drugs for at least 5 years, with better overall survival for patients on drugs compared to those on drugs for less than 5 years. There was no statistical difference between RFS(χ^2^ = 1.572, *P*=0.210) and OS(χ^2^ = 2.725, *P*=0.099). Of the 349 patients using endocrine medication in the low CONUT subgroup, 113 used medication for less than 5 years and 236 patients used endocrine medication for no less than 5 years. The analysis concluded that patients with longer duration of drug use had better survival compared to those with shorter duration of use. No statistical difference between RFS(χ^2^ = 0.307, *P*=0.579) and OS(χ^2^ = 3.573, *P*=0.059). The above analysis concluded that patients who had been on endocrine drugs for a longer period had relatively better overall and relapse-free survival in both the high CONUT and low CONUT subgroups.

**Figure 6:**
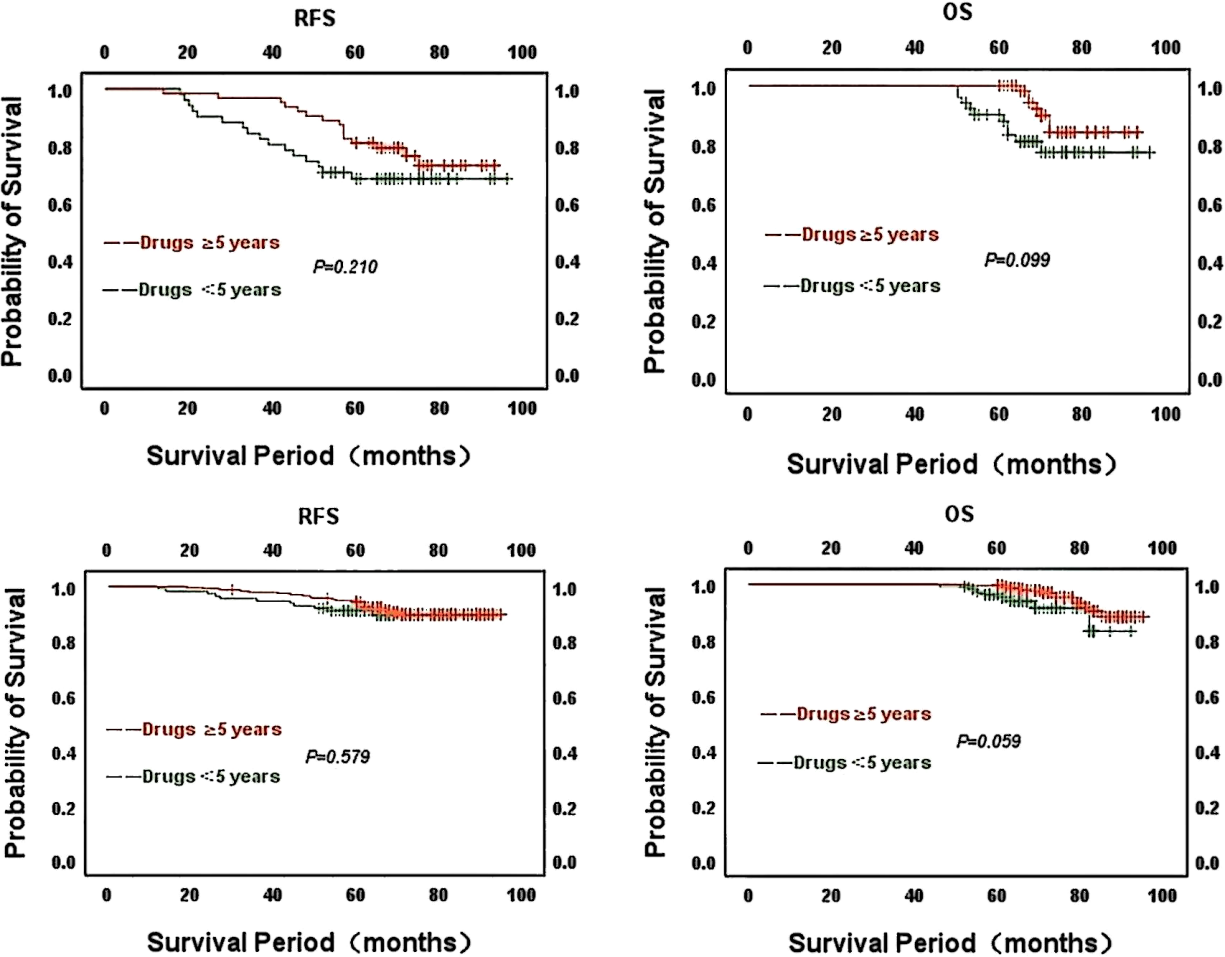
Kaplan-Meier survival curves for the duration of endocrine drug use in patients with strong ER positivity in different CONUT score groups Above: CONUT high group; Below: CONUT low group.

Kaplan-Meier survival curves to statistically analyze the duration of endocrine drug use in patients in different CONUT score groups. Of the 114 patients using endocrine drugs in the CONUT high subgroup, 63 had been on endocrine drugs for at least 5 years. The analysis yielded no statistical difference in the effect of the duration of drug use in the CONUT high group on patients’ RFS(χ^2^ = 1.572, *P*=0.210) and OS(χ^2^ = 2.725, *P*=0.099). Of the 349 patients using endocrine drugs in the low CONUT subgroup, 113 had been using the drugs for less than 5 years and 236 patients had been using endocrine drugs for no less than 5 years. There was also no statistical difference in RFS (χ^2^ = 0.307, *P*=0.579) and OS(χ^2^ = 3.573, *P*=0.059) for patients by the length of drug use in the lower subgroups. However, the curves show that patients with longer duration of endocrine drug use had relatively better overall survival and relapse-free survival in both the CONUT high and CONUT low subgroups (**Figure 6**).

### The predictive role of CONUT scores in HER2-positive patients

#### Relationship between CONUT scores and patient characteristics at baseline

The median age of patients was 55 years, 124 (61.4%) were not younger than 55 years and 155 (76.7%) had a history of breastfeeding before diagnosis; 13.9% of breast cancer patients had no children before diagnosis; 50.5% of breast cancer patients were menopausal at diagnosis; about 38% of breast cancer patients were overweight; The positive rate of lymph nodes was 51.0% (103/202); postoperative TNM staging, 43 (21.3%) patients with stage I, 109 (54.0%) patients with stage II and 50 (24.7%) patients with stage III; 144 (71.3%) patients positive for ER and 122 (60.4%) patients positive for PR. The baseline characteristics of breast cancer patients are shown in **Supplementary Table 1**.

#### Relationship between CONUT score and survival in HER2-positive breast cancer patients

The median follow-up time for the whole group was 72 months (range: 12-98 months), with a total of 45 cases (22.2%) of recurrent metastases during the follow-up period and RFS rates of 88.2%, 76.8%, and 71.4% at 3, 5 and 7 years, respectively. The 5-year RFS rates were 66.7% and 80.3%, respectively, with statistically significant differences (χ^2^ = 5.455, *P*=0.020). **Supplementary Image 1**

Univariate and multifactorial analyses of factors affecting RFS showed that lymph node metastasis (HR=7.900, 95% CI=3.315-18.829, *P*<0.001), tumor size (HR=5.331, 95% CI=2.083-13.641, *P*<0.001) and CONUT score (HR=2.127, 95% CI= 1.153-3.925, *P*=0.016) were independent predictors of recurrent metastasis in patients with HER2-positive early to mid-stage breast cancer receiving regular treatment, see **Supplementary Table 2**.

The Kaplan-Meier curve demonstrated a significantly higher rate of recurrence and metastasis in the high CONUT group compared to the low CONUT group, with 5-year RFS rates of 91.7% and 78.3% respectively, a statistically significant difference (χ^2^ = 5.455, *P*=0.020). The difference was statistically significant (χ^2^ = 5.455, *P*=0.020).

Univariate and multifactorial analyses of factors affecting OS showed that lymph node metastasis (HR=5.822, 95% CI=1.687-20.093, *P*=0.005), tumor size (HR=9.292, 95% CI=1.230-70.197, *P*=0.031) and CONUT score (HR=2.127, 95%CI= 1.153-3.925, *P*=0.016) were independent predictors of overall survival in patients with HER2-positive early to mid-stage breast cancer receiving regular treatment, see **Supplementary Table 3**.

## Discussions

Breast cancer is a heterogeneous disease and precise treatment is chosen according to the individual patient’s condition. In recent years, there is increasing evidence that the immune-nutritional status of the body plays an important role in tumor development and treatment tolerance. The CONUT score reflects the nutritional status of patients and the immune defense function of the body through the combination of peripheral lymphocyte count, serum albumin and total cholesterol level. Similarly, tumor progression and treatment tolerance are closely related to nutritional and inflammatory status (23).

Daisuke et al. (24) demonstrated that CONUT can be used not only to assess the nutritional status of patients with gastric cancer and help guide the selection of appropriate preoperative nutritional interventions but also to predict long-term OS after radical gastrectomy for gastric cancer. Yuki Nemoto et al. (25) studied the effect of CONUT score on tumor outcome after radical cystectomy for advanced bladder cancer and showed that recurrence-free survival, specific survival, and overall survival were significantly shorter in the high CONUT group than in the low CONUT group. Huang et al. (26) also studied and discussed the correlation between CONUT scores and breast. Yi et al. used the CONUT score in a predictive study of HER2-positive breast cancer and showed that the CONUT score was an independent risk factor for recurrence and metastasis in patients with HER2-positive early breast cancer.

Based on previous studies, the CONUT score has shown some promise for clinical application. Our study focused on a unique subtype of all breast cancers for data analysis, which has unique pathological features and biological characteristics and is the most represented molecular subtype of all breast cancer patients, and it is more clinically relevant to understand the association of CONUT scores with this type of breast cancer. Therefore, this study retrospectively collected patients with surgically treated HER2 low-expression breast cancer to assess the relationship between CONUT scores and recurrence, metastasis, and survival in patients with this particular type of breast cancer.

Our study showed that higher CONUT scores correlated with tumor size, lymph node infiltration, and blood markers CEA and CA153 in all patients with low HER2 expression. Among the pathological variables, we found higher CONUT scores with higher Ki-67 status apparent value-added scores. Analysis of univariate and multifactorial Cox proportional hazards regression models showed that the CONUT score was an independent prognostic factor for OS and RFS. Our results are similar to those of previous studies (27), with patients with lower CONUT scores exhibiting longer RFS and OS compared to the high CONUT subgroup. but our study was conducted in a separate HER2 subtype, which not only brings more nuance to breast cancer but is also an important complementary part of the role of such predictors in breast cancer. At the same time, the role of the CONUT score in HER2-positive subtypes was further analyzed in our additional data and the results are similar to previous studies, confirming that the CONUT score is an independent prognostic indicator for this type of breast cancer and reinforcing the use of the CONUT score in the prognosis of HER2 subtypes of breast cancer. High CONUT scores not only suggest a high risk of recurrence and metastasis but also suggest relatively poor overall survival for patients.

Considering the possible influence of ER expression on this type, we compared the correlation between patients with strong positive ER and CONUT scores, but the chi-square test showed no statistically significant difference. Comparing the association with the use of endocrine drugs and the different CONUT score groups showed that patients using endocrine drugs had better survival than those not using endocrine drugs, regardless of whether they were in the high or low group. Patients who used endocrine drugs for more than 5 years had better survival than those who used them for less than 5 years. We also explored the predictive role of the CONUT score in HER2-positive patients. The results were similar to those in HER2 low expressing breast cancer, with the CONUT high subgroup having worse RFS and OS than the CONUT low subgroup.

Among the 3 evaluation parameters of the CONUT score, serum albumin level is the most important influencing factor. It not only reflects the nutritional status of patients but also correlates with the incidence of postoperative complications, mortality and overall survival of oncology patients (28–31). A decrease in serum albumin weakens cellular and humoral immunity, phagocytosis, and other defense mechanisms in cancer patients, leading to an inflammatory response, and conversely, serum albumin levels are reduced by inflammation (32, 33). Many studies have confirmed that low serum albumin levels predict poorer morbidity and mortality in solid and hematologic tumors (34). As the most commonly used inflammatory indicator in the clinic, peripheral blood lymphocytes play a crucial role in tumor immunity, such as cytotoxic cell death, tumor cell proliferation, migration inhibition, etc. Its decrease reflects the suppression of the body’s tumor immune function, which puts the growth of tumor cells in an infinite proliferation state, which in turn leads to tumor metastasis and poor prognosis (35–38). Likewise, cholesterol is an essential lipid for maintaining cellular homeostasis and is involved in the acquired and adaptive immune responses of the body, playing a critical role in cell membrane formation and various biochemical pathways that are essential for normal biological functions. The relationship between total serum cholesterol levels and tumorigenesis, prognostic outcome, and chemotherapy resistance has been reported in various malignancies (39–41).

The above evidence may explain the relationship between CONUT scores and the poor prognosis of tumor patients. The results of this paper and the above studies suggest that the CONUT score may also be an important indicator for the prognosis of breast cancer patients and that the three categories of blood indicators included in the CONUT score can reflect the immune-nutritional status of individuals more accurately and comprehensively than previous predictors, helping clinicians to accurately identify patients at high risk and thus provide more individualized and comprehensive management plans for cancer patients. This study has some limitations in that the patient sample size was limited and the data collected were from a single-center retrospective pilot study of only one specific type of breast cancer patient, which increases the risk of bias. At the same time, this article lacks external data to validate the cohort, and the prognostic value of the CONUT score for breast cancer patients with different molecular subtypes still needs to be confirmed by large, multicenter, prospective studies.

## Conclusion

The preoperative CONUT score may be a useful predictor of postoperative recurrence and metastasis in patients with HER2-low breast cancer, monitoring oncology patients and providing clinical guidance for more individualized treatment.

## Data availability

The raw data supporting the conclusions of this article will be made available by the authors, without undue reservation.

## Ethics statement

The studies involving human participants were reviewed and approved by Ethics Committee of Harbin Medical University Cancer Hospital. All procedures in this study comply with the Commission’s standards, as well as the 2013 Declaration of Helsinki and other ethical standards. The patients/participants provided their written informed consent to participate in this study.

## Author contributions

YL and YZ: Conceptualization, Data curation, Formal analysis, Writing-original draft. ZZ: Data curation, Formal analysis. LS: Data curation, Formal analysis. YH: Data curation, Formal analysis. XL: Data curation, Formal analysis. SC: Data curation, Formal analysis, Writing-review & editing. All authors contributed to the article and approved the submitted version.

## Supplementary Material

Supplementary Image 1 Kaplan-Meier survival curves for recurrence-free survival and overall survival in HER2-positive breast cancer with CONUT score.
