# Supplementary figures and images for: Predictive value of controlling nutritional status score in postoperative recurrence and metastasis of breast cancer patients with HER2-low expression
Source: Front Oncol. 2023 Jul 10;13:1116631. doi: 10.3389/fonc.2023.1116631 (PMC10365291; doi:10.3389/fonc.2023.1116631)

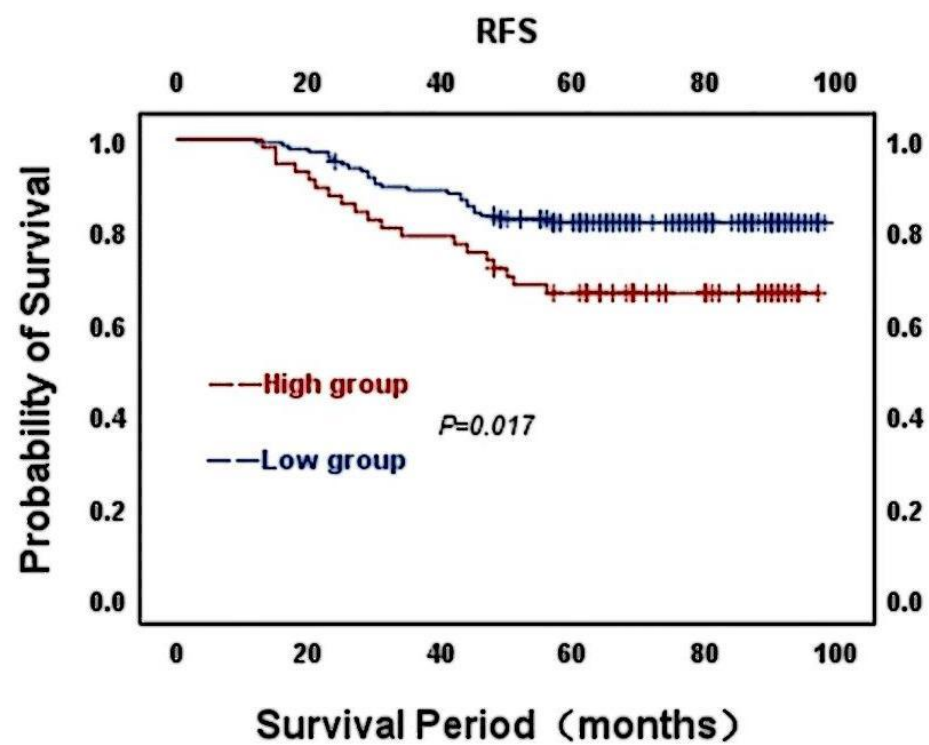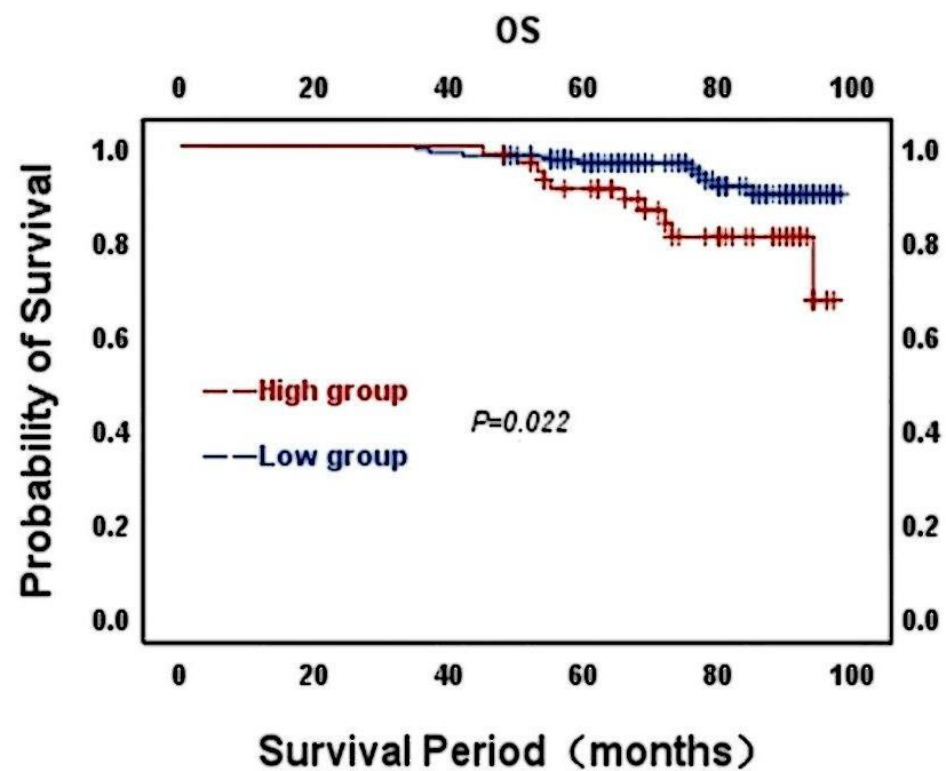

Supplement: Supplementary Image 1 — Kaplan-Meier survival curves for recurrence-free survival and overall survival in HER2-positive breast cancer with CONUT score. [file Image_1.pdf]
