# Supplementary material for: Predictive value of controlling nutritional status score in postoperative recurrence and metastasis of breast cancer patients with HER2-low expression
Source: Front Oncol. 2023 Jul 10;13:1116631. doi: 10.3389/fonc.2023.1116631 (PMC10365291; doi:10.3389/fonc.2023.1116631)
Supplement: Supplementary file 2 [file Table_1.docx]

Schedule 1. Baseline characteristics of patients in the low CONUT group and the high CONUT group

| Characteristics | Total (n=202) | CONUT SCORE | | χ^2^/Fisher | *p-*value |
| --- | --- | --- | --- | --- | --- |
|  |  | Low(n=145) | High(n=57) |  |  |
| Age(years) |  |  |  | 29.764 | **<**0.001 |
| １<55 | 78(38.6%) | 39(19.3%) | 39(19.3%) |  |  |
| １≥55 | 124(61.4%) | 106(52.5%) | 18(8.9%) |  |  |
| Reproductive history |  |  |  | 1.723 | 0.189 |
| １Yes | 174(86.1%) | 122(60.4%) | 52(25.7%) |  |  |
| １No | 28(13.9%) | 23(11.4%) | 5(2.5%) |  |  |
| Breastfeeding history |  |  |  | 0.009 | 0.923 |
| １Yes | 155(76.7%) | 111(55.0%) | 44(21.7%) |  |  |
| １No | 47(23.3%) | 34(16.8%) | 13(6.5%) |  |  |
| Menopause status |  |  |  | 0.060 | 0.807 |
| １Yes | 102(50.5%) | 74(36.6%) | 28(13.9%) |  |  |
| １No | 100(49.5%) | 71(35.1%) | 29(14.4%) |  |  |
| BMI (kg/m^2^) |  |  |  | 1.287 | 0.565 |
| １<25 | 127(62.9%) | 90(44.6%) | 37(18.3%) |  |  |
| １25-30 | 65(33.0%) | 49(24.3%) | 16(7.9%) |  |  |
| １≥30 | 10(5.0%) | 6(3.0%) | 4(2.0%) |  |  |
| CEA (ng/mL) |  |  |  | 1.186 | 0.276 |
| 1Negative | 164(81.2%) | 115(56.9%) | 49(24.3%) |  |  |
| 1Positive | 38(18.8%) | 30(14.9%) | 8(3.9%) |  |  |
| CA153 |  |  |  | 0.011 | 0.917 |
| 1Negative | 180(89.1%) | 129(63.9%) | 51(25.2%) |  |  |
| 1Positive | 22(10.9%) | 16(7.9%) | 6(3.0%) |  |  |
| Surgery type |  |  |  | 0.030 | 0.862 |
| １BCS | 39(19.3%) | 25(12.4%) | 14(6.9%) |  |  |
| １Mastectomy | 163(80.7%) | 120(59.4%) | 43(21.3%) |  |  |
| KI67 status |  |  |  | 0.100 | 0.752 |
| １≤14% | 64(31.7%) | 45(22.3%) | 19(9.4%) |  |  |
| １>14% | 138(68.3%) | 100(49.5%) | 38(18.8%) |  |  |
| ER status |  |  |  | 0.669 | 0.414 |
| １Negative | 58(28.7%) | 44(21.8%) | 14(6.9%) |  |  |
| １Positive | 144(71.3%) | 101(50.0%) | 43(21.3%) |  |  |
| PR status |  |  |  | 0.253 | 0.615 |
| １Negative | 80(39.6%) | 59(29.2%) | 21(10.4%) |  |  |
| １Positive | 122(60.4%) | 86(42.6%) | 36(17.8%) |  |  |
| pT Stage |  |  |  | 1.666 | 0.449 |
| １1 | 76(37.6%) | 58(28.7%) | 18(8.9%) |  |  |
| １2 | 121(59.9%) | 84(41.6%) | 37(18.3%) |  |  |
| １3 | 5(2.5%) | 3(1.5%) | 2(1.0%) |  |  |
| pN stage |  |  |  | 2.294 | 0.521 |
| １0 | 99(49.0%) | 70(34.7%) | 29(14.3%) |  |  |
| 111 | 56(27.7%) | 43(21.3%) | 13(6.4%) |  |  |
| 212 | 33(16.3%) | 24(11.8%) | 9(4.5%) |  |  |
| １3 | 14(6.9%) | 8(3.9%) | 6(3.0%) |  |  |
| pTNM stage |  |  |  | 0.495 | 0.833 |
| １Ⅰ | 43(21.3%) | 31(15.3%) | 12(6.0%) |  |  |
| １Ⅱ | 109(54.0%) | 80(39.6%) | 29(14.4%) |  |  |
| １Ⅲ | 50(24.7%) | 34(16.8%) | 16(7.9%) |  |  |
| Chemotherapy |  |  |  | 0.073 | 0.788 |
| １Yes | 148(73.3%) | 107(53.0%) | 41(20.3%) |  |  |
| １No | 54(26.7%) | 38(18.8%) | 16(7.9%) |  |  |
| Endocrine therapy |  |  |  | 3.0.278 | 0.598 |
| １Yes | 111(55.0%) | 78(38.7%) | 33(16.3%) |  |  |
| １No | 91(45.0%) | 67(33.2%) | 24(11.8%) |  |  |
| Targeted therapy |  |  |  | 2.751 | 0.097 |
| １Yes | 51(25.2%) | 32(15.8%) | 19(9.4%) |  |  |
| １No | 151(74.8%) | 113(55.9%) | 38(18.9%) |  |  |
