# Supplementary material for: Predictive value of controlling nutritional status score in postoperative recurrence and metastasis of breast cancer patients with HER2-low expression
Source: Front Oncol. 2023 Jul 10;13:1116631. doi: 10.3389/fonc.2023.1116631 (PMC10365291; doi:10.3389/fonc.2023.1116631)
Supplement: Supplementary file 3 [file Table_2.docx]

Schedule 2. Univariate and multivariate analyses of recurrence free survival

| Parameters | Univariate analysis |  | Multivariate analysis |  |
| --- | --- | --- | --- | --- |
|  | Hazard ratio(95%CI) | *P* value | Hazard ratio(95%CI) | *P* value |
| Age (years) |  | 0.399 |  |  |
| 1<55 | 1(reference) |  |  |  |
| 1≥55 | 1.306(0.702-2.427) |  |  |  |
| BMI (kg/m^2^) |  | 0.068 |  |  |
| 1<25 | 1(reference) |  |  |  |
| 1≥25 | 1.722(0.960-3.091) |  |  |  |
| CEA |  | 0.306 |  |  |
| 1Negative | 1(reference) |  |  |  |
| 1Positive | 0.638(0.270-1.508) |  |  |  |
| CA153 |  | 0.614 |  |  |
| 1Negative | 1(reference) |  |  |  |
| 1Positive | 0.768(0.275-2.143) |  |  |  |
| ER status |  | 0.437 |  | 0.438 |
| 1Negative | 1(reference) |  | 1(reference) |  |
| 1Positive | 0.782(0.421-1.453) |  | 1.389(0.606-3.182) |  |
| PR status |  | 0.269 |  | 0.079 |
| 1Negative | 1(reference) |  | 1(reference) |  |
| 1Positive | 0.719(0.400-1.291) |  | 0.490(0.220-1.088) |  |
| KI-67 |  | 0.408 |  | 0.405 |
| 1<14% | 1(reference) |  | 1(reference) |  |
| 1≥14% | 0.775(0.424-1.416) |  | 0.769(0.413-1.429) |  |
| Tumor size |  | <0.001 |  | <0.001 |
| 1≤2 | 1(reference) |  | 1(reference) |  |
| 1>2 | 5.780(2.280-14.651) |  | 5.331(2.083-13.641) |  |
| Lymphatic metastasis |  | <0.001 |  | <0.001 |
| 1No | 1(reference) |  | 1(reference) |  |
| 1Yes | 7.725(3.268-18.263) |  | 7.900(3.315-18.829) |  |
| CONUT score |  | 0.020 |  | 0.016 |
| 1<3 | 1(reference) |  | 1(reference) |  |
| 1≥3 | 2.023(1.120-3.656) |  | 2.127(1.153-3.925) |  |
