# Supplementary material for: Predictive value of controlling nutritional status score in postoperative recurrence and metastasis of breast cancer patients with HER2-low expression
Source: Front Oncol. 2023 Jul 10;13:1116631. doi: 10.3389/fonc.2023.1116631 (PMC10365291; doi:10.3389/fonc.2023.1116631)
Supplement: Supplementary file 4 [file Table_3.docx]

Schedule 3. Univariate and multivariate analyses of overall survival

| Parameters | Univariate analysis |  | Multivariate analysis |  |
| --- | --- | --- | --- | --- |
|  | Hazard ratio(95%CI) | *P* value | Hazard ratio(95%CI) | *P* value |
| Age (years) |  | 0.479 |  |  |
| 1<55 | 1(reference) |  |  |  |
| 1≥55 | 0.731(0.307-1.741) |  |  |  |
| BMI (kg/m^2^) |  | 0.865 |  |  |
| 1<25 | 1(reference) |  |  |  |
| 1≥25 | 1.080(0.445-2.617) |  |  |  |
| CEA |  | 0.470 |  |  |
| 1Negative | 1(reference) |  |  |  |
| 1positive | 0.636(0.187-2.167) |  |  |  |
| CA153 |  | 0.784 |  |  |
| 1Negative | 1(reference) |  |  |  |
| 1Positive | 0.815(0.190-5.503) |  |  |  |
| ER status |  | 0.122 |  | 0.640 |
| 1Negative | 1(reference) |  | 1(reference) |  |
| 1Positive | 0.505(0.213-1.200) |  | 0.747(0.221-2.533) |  |
| PR status |  | 0.311 |  | 0.456 |
| 1Negative | 1(reference) |  | 1(reference) |  |
| 1Positive | 0.624(0.272-1.513) |  | 0.623(0.180-2.162) |  |
| KI-67 |  | 0.326 |  | 0.284 |
| 1<14% | 1(reference) |  | 1(reference) |  |
| 1≥14% | 0.648(0.273-1.539) |  | 0.612(0.249-1.503) |  |
| Tumor size |  | 0.012 |  | 0.031 |
| 1≤2 | 1(reference) |  | 1(reference) |  |
| 1>2 | 12.945(1.737-96.469) |  | 9.292(1.230-70.197) |  |
| Lymphatic metastasis |  | 0.004 |  | 0.005 |
| 1No | 1(reference) |  | 1(reference) |  |
| 1Yes | 6.175(1.818-20.971) |  | 5.822(1.687-20.093) |  |
| Postoperative chemotherapy |  | 0.709 |  |  |
| 1No | 1(reference) |  |  |  |
| 1Yes | 1.211(0.442-3.318) |  |  |  |
| Postoperative endocrinetherapy |  | 0.745 |  |  |
| 1No | 1(reference) |  |  |  |
| 1Yes | 0.868(0.368-2.044) |  |  |  |
| CONUT score |  | 0.013 |  | 0.006 |
| 1<3 | 1(reference) |  | 1(reference) |  |
| 1≥3 | 2.967(1.259-6.990) |  | 3.480(1.422-8.516) |  |
